# Inhibition of leukocyte migration after ischemic stroke by VE‐cadherin mutation in a mouse model leads to reduced infarct volumes and improved motor skills

**DOI:** 10.1002/brb3.3449

**Published:** 2024-03-12

**Authors:** Mailin Hannah Marie Koecke, Jan‐Kolja Strecker, Frederike Anne Straeten, Carolin Beuker, Jens Minnerup, Antje Schmidt‐Pogoda, Anna‐Lena Börsch

**Affiliations:** ^1^ Department of Neurology with Institute of Translational Neurology University of Münster Münster Germany

**Keywords:** ischemic cascade, ischemic stroke, knock‐in mice, leukocyte migration, MCAO, vascular permeability, VE‐cadherin mutations

## Abstract

**Aims:**

To distinguish between the genuine cellular impact of the ischemic cascade by leukocytes and unspecific effects of edema and humoral components, two knock‐in mouse lines were utilized. Mouse lines Y731F and Y685F possess point mutations in VE‐cadherin, which lead to a selective inhibition of transendothelial leukocyte migration or impaired vascular permeability.

**Methods:**

Ischemic stroke was induced by a model of middle cerebral artery occlusion. Analysis contained structural outcomes (infarct volume and extent of brain edema), functional outcomes (survival analysis, rotarod test, and neuroscore), and the extent and spatial distribution of leukocyte migration (heatmaps and fluorescence‐activated cell sorting (FACS) analysis).

**Results:**

Inhibition of transendothelial leukocyte migration as in Y731F mice leads to smaller infarct volumes (52.33 ± 4719 vs. 70.43 ± 6483 mm^3^, *p* = .0252) and improved motor skills (rotarod test: 85.52 ± 13.24 s vs. 43.06 ± 15.32 s, *p* = .0285). An impaired vascular permeability as in Y685F mice showed no effect on structural or functional outcomes. Both VE‐cadherin mutations did not influence the total immune cell count or spatial distribution in ischemic brain parenchyma.

**Conclusion:**

Selective inhibition of transendothelial leukocyte migration by VE‐cadherin mutation after ischemic stroke in a mouse model leads to smaller infarct volumes and improved motor skills.

## Graphical abstract

Inhibition of leukocyte migration after ischemic stroke by VE‐cadherin mutation in a mouse model leads to reduced infarct volumes and improved motor skills. VE‐cadherin mutation Y731F is associated with a selective inhibition of transendothelial leukocyte migration. In a mouse model of ischemic stroke, it led to smaller infarct volumes and improved motor skills. VE‐cadherin mutation Y685F had no effect on structural or functional outcomes on the same mouse model of ischemic stroke.

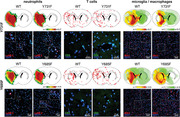

## 1 INTRODUCTION

Migration of immune cells through the blood–brain barrier and their consecutive effects have been identified as one of the major determinants for secondary injury after an ischemic stroke (Iadecola & Anrather, 2011). As part of the post‐ischemic cascade, leukocyte migration causes the release of proinflammatory cytokines, free radicals, and proteolytic enzymes, creating a proinflammatory environment that leads to an increased infarct core and loss of penumbra (Kleinig & Vink, 2009; Moskowitz et al., 2010). Other studies that intended to prevent leukocyte diapedesis into the brain parenchyma yielded conflicting results and showed either the reduction of infarct volumes and improvement of neuroscores (Becker et al., 2001; Neumann et al., 2015) or could not generate significantly differing results (Langhauser et al., 2014). In a multicenter study, the group was confronted with contradicting results within their own findings depending on the middle cerebral artery occlusion (MCAO) model and extension of the induced infarct (Liesz et al., 2011; Llovera et al., 2015). The difficulties with finding a potent therapeutic agent continued in clinical trials (Becker, 2002; Elkins et al., 2017; Enlimomab Acute Stroke Trial Investigators, 2001). These studies either used a depletion model or applied antibodies against adhesion molecules like intercellular adhesion molecule 1 (ICAM‐1), vascular cell adhesion molecule 1 (VCAM‐1), and very late antigen‐4 (VLA‐4).

Here, we used a different approach of modulating the blood–brain barrier permeability using two different knock‐in mouse lines with point mutations in VE‐cadherin. VE‐cadherin plays a crucial role in regulating the permeability of the blood–brain barrier (Gavard, 2014). Phosphorylation and dephosphorylation of specific tyrosine residues of this junctional protein were identified as key players in this process (Vestweber et al., 2014; Wessel et al., 2014). The phosphorylation of tyrosine residue 685 induces inflammation‐associated increased vascular permeability, whereas the dephosphorylation of tyrosine residue 731 enables leukocyte diapedesis (Wessel et al., 2014). Two knock‐in mouse lines were created in which these specific tyrosine residues were exchanged for phenylalanine by point mutation to impair phosphorylation and dephosphorylation. One knock‐in mouse line therefore presented an altered blood–brain barrier permeability for water and macromolecules (Y685F), the other for leukocytes (Y731F) (Gavard, 2014; Vestweber, 2008). By comparing the results of these two mouse lines, we were able to get an idea of the genuine cellular impact of the ischemic cascade caused by leukocytes in contrast to unspecific effects of edema and humoral components.

## 2 METHODS

### 2.1 Transgenic mice

The study was conducted in accordance with national guidelines for the use of experimental animals, and the protocols were approved by the governmental committees (Landesamt für Natur, Umwelt und Verbraucherschutz Nordrhein‐Westfalen). Two knock‐in mouse lines were created carrying two different point mutations in VE‐cadherin: Y685F and Y731F. The heterozygotic mice were created at the Max Planck Institute for Biomedicine in Münster (Director: Prof. Dr. Dietmar Vestweber) and later on mated to generate homozygotic mice at our animal facility.

Y685F mice had the amino acid tyrosine on position 685 replaced by phenylalanine, leading to an impaired possibility of phosphorylation. This caused reduced transendothelial permeability for water and macromolecules compared to their wildtype littermates without an effect on leukocyte diapedesis. Although Y731F mice presented reduced transendothelial permeability for leukocytes compared to their wildtype littermates. Causative was the same amino acid switch on position 731, leading to an impaired possibility of dephosphorylation (Vestweber et al., 2014; Wessel et al., 2014).

### 2.2 Stroke model

Focal transient ischemic stroke was induced through 45 min of transient MCAO, as previously described (Gelderblom et al., 2009; Gliem et al., 2012; Schmidt‐Pogoda et al., 2018; Zhu et al., 2023). In brief, the procedure was performed under inhalation anesthesia with 1.5% isoflurane in 30% O_2_/70% N_2_O and maintenance of a constant body temperature of 37 ± 0.5°C. The left common carotid artery and the carotid bifurcation were exposed, and the proximal common carotid artery and external carotid artery were permanently ligated. To transiently prevent retrograde flow of blood into the left common carotid artery, a microvascular clip (FE691; Aesculap) was used. An incision was placed along the common carotid artery, and a silicon‐coated 8‐0 nylon monofilament (701956PK5Re, Doccol Corporation) was advanced into the middle cerebral artery. Verification of MCAO was performed by laser Doppler (Periflux 5001; Perimed). After 45 min of MCAO, the monofilament was withdrawn to allow reperfusion.

### 2.3 Functional outcome assessment

For functional outcome assessment, we employed a modification of Menzies’ neuroscore (Menzies et al., 1992) (Y731F^d/d^: *n* = 11, Y731F^+/+^: *n* = 10, Y685F^d/d^: *n* = 7, Y685F^+/+^: *n* = 9) and the rotarod test (Y731F^d/d^: *n* = 15, Y731F^+/+^: *n* = 9, Y685F^d/d^: *n* = 8, Y685F^+/+^: *n* = 8) at baseline, 24, 48, and 72 h after ischemic stroke. Further, we analyzed survival. All assessments were performed by a blinded observer.

Based on the modified neuroscore according to Menzies’, animals were scored from 0 (no neurological impairment) to 5 (death). Survival was plotted by means of Kaplan–Meier curves. In the rotarod test, the time the animals could stay on an accelerating rod was measured (Hamm et al., 1994).

### 2.4 Structural outcome assessment

Structural outcome was displayed by infarct volume and the extent of brain edema. The prior determined time points for infarct volumes were 72 h after MCAO (Y731F^d/d^: *n* = 13, Y731F^+/+^: *n* = 6, Y685F^d/d^: *n* = 11, Y685F^+/+^: *n* = 7). In some cases, mice died prematurely after 24 h (Y731F^d/d^: *n* = 0, Y731F^+/+^: *n* = 1, Y685F^d/d^: *n* = 1, Y685F^+/+^: *n* = 3) or 48 h (Y731F^d/d^: *n* = 0, Y731F^+/+^: *n* = 2, Y685F^d/d^: *n* = 0, Y685F^+/+^: *n* = 0) after MCAO. The infarct volumes at all three time points were analyzed together. After the animals were transcardially perfused, their brains were cryoconserved and coronally cut into thin slices of 10 μm at 300 μm intervals. To assess the infarct volume, these cryosections were stained with toluidine blue, which leads to a dark blue staining of vital brain parenchyma, whereas the infarcted area appears white. Later on, photographs of the stained cryosections were taken under a microscope, and the area was calculated with the software *ImageJ*, which contained a correction for cortical edema. All measured areas added up to the infarct volume. Brain edema was displayed as a percentage of the ipsilateral hemisphere (Y731F^d/d^: *n* = 18, Y731F^+/+^: *n* = 12, Y685F^d/d^: *n* = 13, Y685F^+/+^: *n* = 11). Regarding the time points, same applies for the extent of the edema as for the infarct volumes. To calculate the brain edema, the difference between ipsilateral and contralateral hemispheres of all sections in one group was added up and divided by the sum of the ipsilateral hemisphere of all sections. To further assess vascular leakage, the amount of intraparenchymal albumin (Y731F^d/d^: *n* = 6, Y731F^+/+^: *n* = 7, Y685F^d/d^: *n* = 8, Y685F^+/+^: *n* = 8) and IgG (Y731F^d/d^: *n* = 6, Y731F^+/+^: *n* = 7, Y685F^d/d^: *n* = 6, Y685F^+/+^: *n* = 6) were quantified via immunohistochemistry 72 h after MCAO.

### 2.5 Analysis of spatial leukocyte migration

To visualize neutrophils, macrophages/microglia, and T‐lymphocytes, immunohistochemistry was performed on coronal cryosections of animals perfused 72 h after MCAO (*n* = 6). The antibody Iba‐1 was used for macrophages/microglia, Ly6B for neutrophils, and CD3 for T‐cells. The spatial distribution of the migrated immune cells was then analyzed under a fluorescence microscope. For the illustration of the migration process, the distribution of each specific cell was manually transferred to a template of a murine brain in the coronar plane. The area of distribution was subsequently digitally mapped with the software *Adobe Illustrator*, and all areas were fused into one heatmap for one certain group and cell type.

### 2.6 Isolation of CNS‐resident leukocytes

Animals were intracardially perfused with cold phosphate buffered saline under deep ketamine/xylazine anesthesia. The brain tissue was cut into pieces and digested with collagenase D (2.5 mg/mL, Roche Diagnostics) and DNase I (0.05 mg/mL, Sigma) at 37°C for 20 min. Subsequently, digested tissue was passed through a 70 μm cell strainer, and brain cells were centrifuged on a 70%/37% Percoll gradient. The interphase was collected, washed, and resuspended in phosphate‐buffered saline with 2% fetal calf serum.

### 2.7 Flow cytometry and sorting

The following fluorochrome‐labeled antibodies were used: CD45 (clone 30‐11 F) A700, CD11b (clone M1/70) BV510, Ly‐6C (clone HK1.4) PE, and MHC II (clone M5/114.15.2) BV421, all obtained from BioLegend, as well as Ly‐6G (clone 1A8) FITC obtained from BD Pharmingen and F4/80 (clone BM8) APC obtained from eBioscience. After staining, cells were washed twice and resuspended in phosphate‐buffered saline with 2% fetal calf serum. Cells were acquired on a Gallios flow cytometer (Beckman Coulter) or sorted on a FACS Aria III (BD). Sorting was performed using an 85 μm nozzle and four‐way purity sort precision mode. Data were analyzed using FlowJo software v10.6.1 (BD). Cell concentrations from all tissues were manually counted in a Fuchs‐Rosenthal counting chamber (Y731F^d/d^: *n* = 4, Y731F^+/+^: *n* = 3, Y685F^d/d^: *n* = 4, Y685F^+/+^: *n* = 4).

### 2.8 Sample size calculation

The animal number for each group was determined by a sample size calculation. It was based on the results of previous studies and performed using the sample size calculator available at https://www.stat.ubc.ca/~rollin/stats/ssize/n2.html. The duration animals could stay on a rotarod was selected as the primary endpoint. In preliminary work, an average duration of 70 s was determined. With an expected improvement of 20% to 84 s in animals with the VE‐cadherin mutation and assuming a significance level of *α* = 0.05, this resulted in a test strength (1‐β) of 0.8 (80%) with a number of animals of *n* = 12 per group examined.

## 3 RESULTS

### 3.1 Selective inhibition of transendothelial leukocyte migration improves motor skills and reduces infarct volumes

**FIGURE 1:**
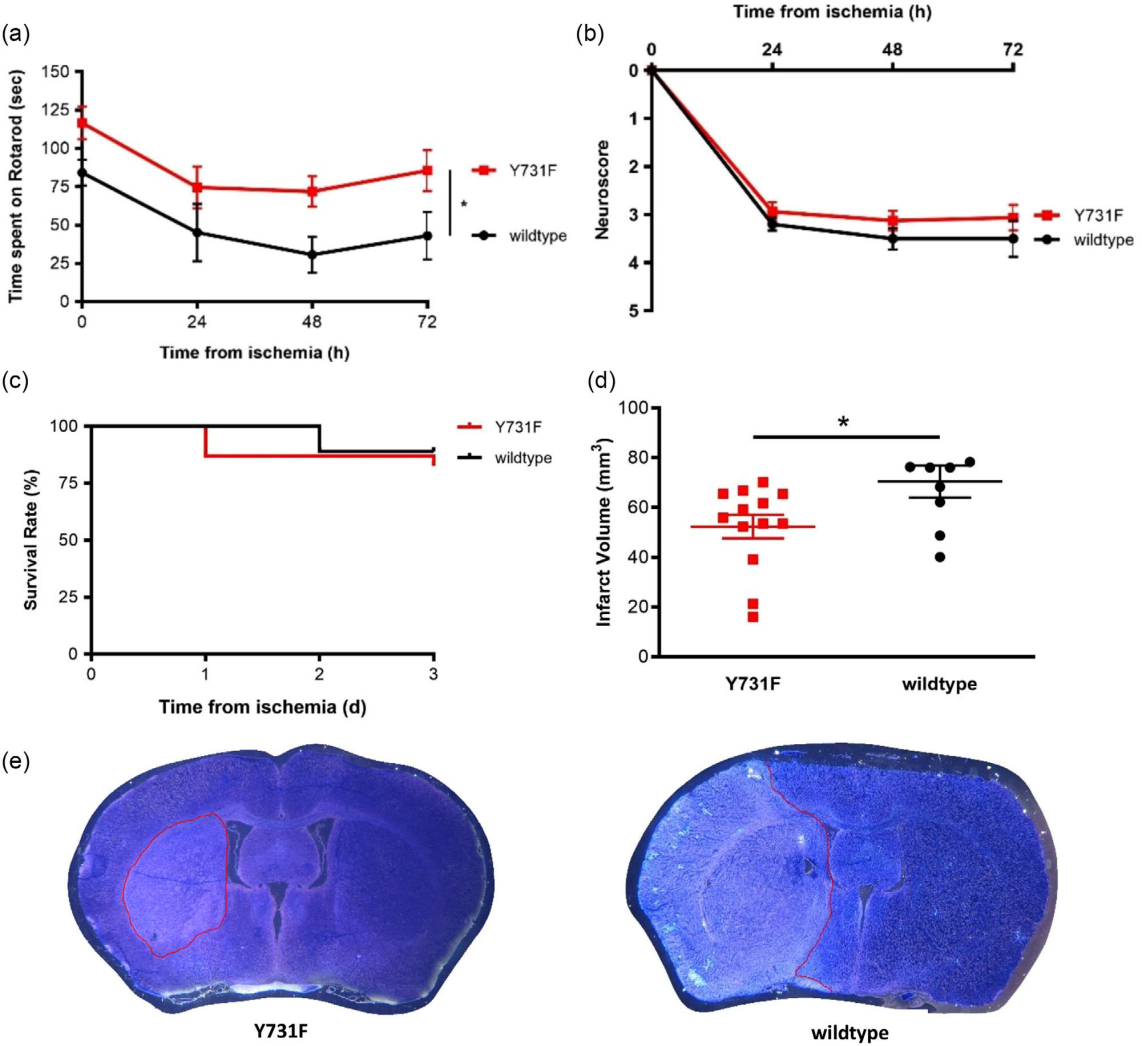
Selective inhibition of transendothelial leukocyte migration improves motor skills and reduces infarct volumes. All results are displayed as mean ± SEM. (a) Rotarod test—results are presented as mean time spent on rotarod in seconds. The Y731F mice (*n* = 15) showed significantly better results on the rotarod than their wildtype littermates (*n* = 9) (two‐way ANOVA, *p < .05, *p* = .0285) on day 3 after middle cerebral artery occlusion (MCAO). (b) Neuroscore—the neuroscore showed a trend toward improved outcome in Y731F mice (*n* = 11 and 10, two‐way ANOVA, *p* = .0792) 72 h after MCAO. (c) Mortality—depicted using Kaplan–Meier curves; there was no difference in the survival rate between Y731F mice (*n* = 23) with a inhibited leukocyte diapedesis and their wildtype littermates (*n* = 16) (logrank test [Mantel–Cox test] and exact Fisher test, *p* = .6544). (d) Infarct volume—Y731F mice (*n* = 13) had significantly smaller infarct volumes compared to their wildtype littermates without the point mutation in VE‐cadherin (*n* = 9) (*t*‐test, *p < .05, *p* = .0313). The unit of measurement of our results is mm^3^. Results are a data compilation of animals that died 24, 48, and 72 h after MCAO. (e) Toluidine staining—representative images of one of the Y731Fd/d mice and one of the wildtype littermates to visually compare the infarct volume. The infarcted area is encircled in red. The infarcted area of the Y731Fd/d mouse appears much smaller than the one of the Wildtype littermate.

To evaluate the impact of a selective inhibition of transendothelial leukocyte migration after ischemic stroke, Y731F mice were compared to their wildtype littermates. Y731F mice showed significantly better results in the rotarod test (Figure 1A; two‐way ANOVA, *p < .05, *p* = .0285, *n* = 15 vs. *n* = 9). The neuroscore showed a trend toward improved outcomes in Y731F mice (Figure 1B; two‐way ANOVA, *p* = .0792, *n* = 16 vs. *n* = 10). The survival analysis (Figure 1C; logrank test [Mantel–Cox test] and exact Fisher test, *p* = .6544, *n* = 23 vs. *n* = 16) did not show a difference between both groups. These findings indicate that the inhibition of leukocyte diapedesis had a distinct impact on motor skills despite a comparable general state of health.

**FIGURE 2:**
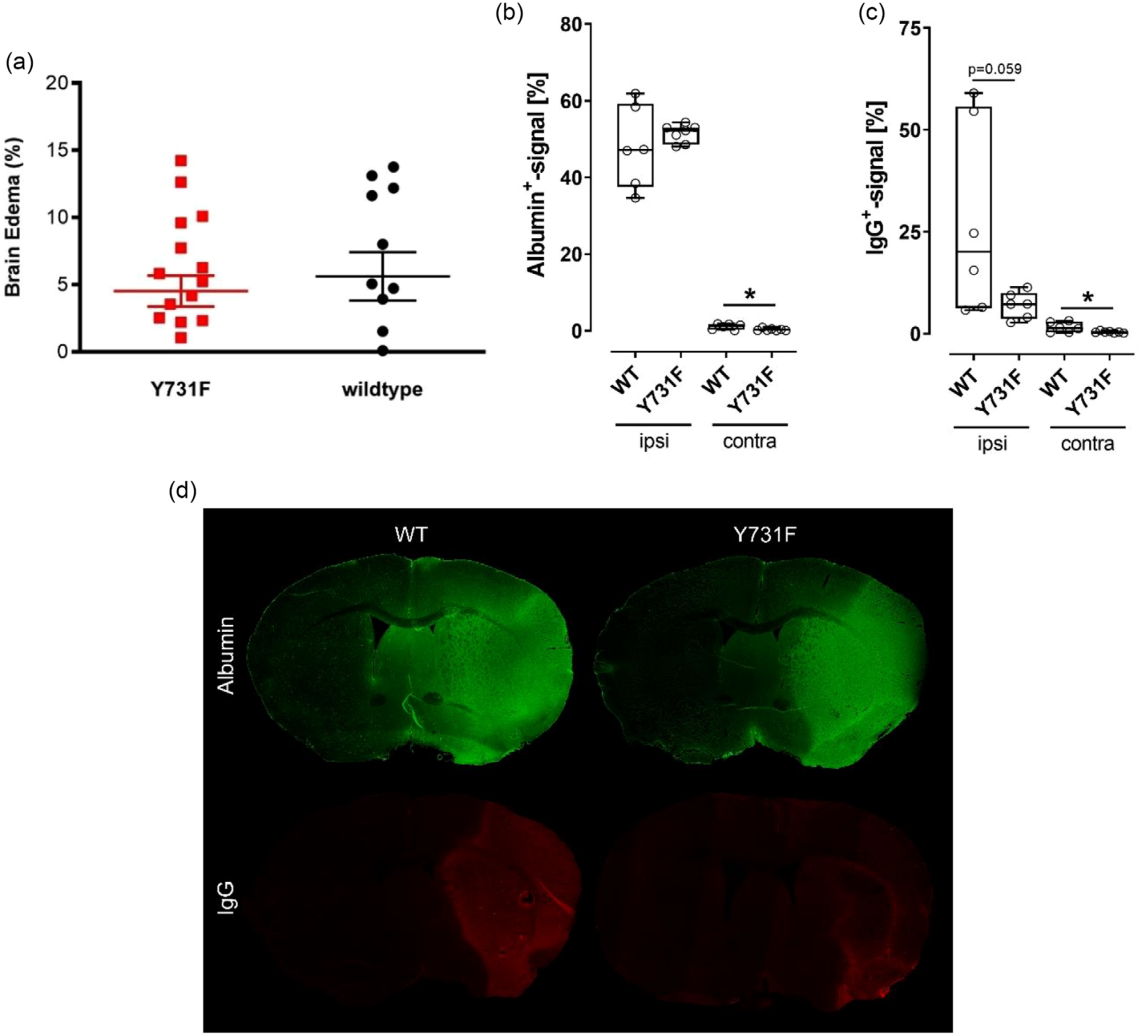
VE‐cadherin mutation Y731F has no influence on the extent of brain edema but leads to reduced IgG leakage. (a) Extent of brain edema—the altered blood–brain barrier permeability for leukocytes had no effect on the extent of brain edema (*n* = 18 and 12, *t*‐test, *p* = .5963). Results are displayed as percentage of the ipsilateral hemisphere. Results are a data compilation of animals that died 24, 48, and 72 h after middle cerebral artery occlusion (MCAO). (b) Quantification of albumin leakage—there was no difference regarding the percentage of albumin signaling in the infarcted hemisphere between Wildtype and Y731F mice (*n* = 6–7, *t*‐test, *p* = .414). In the contralateral hemisphere, the difference was significant with a reduced albumin leakage in Y731F mice (*n* = 6–7, *t*‐test, *p* = .023). The data were collected 72 h after MCAO. (c) Quantification of IgG leakage—there was a tendency toward reduced IgG leakage in the infarcted hemisphere as well as a significantly reduced IgG leakage in the contralateral hemisphere of Y731F mice (*n* = 6–7, *t*‐test, ipsilateral: *p* = .059, contralateral: *p* = .031). The data were collected 72 h after MCAO. (d) Immunohistochemical visualization of albumin and IgG leakage—it also arose the visual impression of reduced IgG leakage in Y731F mice.

Not only the functional outcome but also the structural outcome were measurably affected by the selective inhibition of the transendothelial leukocyte migration. The Y731F mice displayed significantly reduced infarct volumes (Figure 1D; *t*‐test, *p < .05, *p* = .0313, *n* = 13 vs. *n* = 9) compared to their wildtype littermates, whereas the extent of brain edema remained unaffected by the altered blood–brain barrier permeability for leukocytes (Figure 2A; *t*‐test, *p* = .5963, *n* = 18 vs. *n* = 12). Y731F mice presented no difference in albumin leakage regarding the infarcted hemisphere when compared to their wildtype littermates (Figure 2B; *t*‐test, *p* = .414, *n* = 6 vs. *n* = 7). IgG leakage displayed a trend toward a reduction in the infarcted hemisphere and a significant reduction contralateral (Figure 2C; *t*‐test, *p < .05, ipsilateral: *p* = .059, contralateral: *p* = .031, *n* = 6 vs. *n* = 7). These results were supported by the visual impression that arose from the immunohistochemical stainings (Figure 2D). Altogether, these findings suggest that leukocytes are key players in acute stroke pathophysiology, influencing both the functional outcome and the infarct volume.

### 3.2 Reduced permeability for water and macromolecules has no impact on functional and structural outcomes after ischemic stroke

**FIGURE 3:**
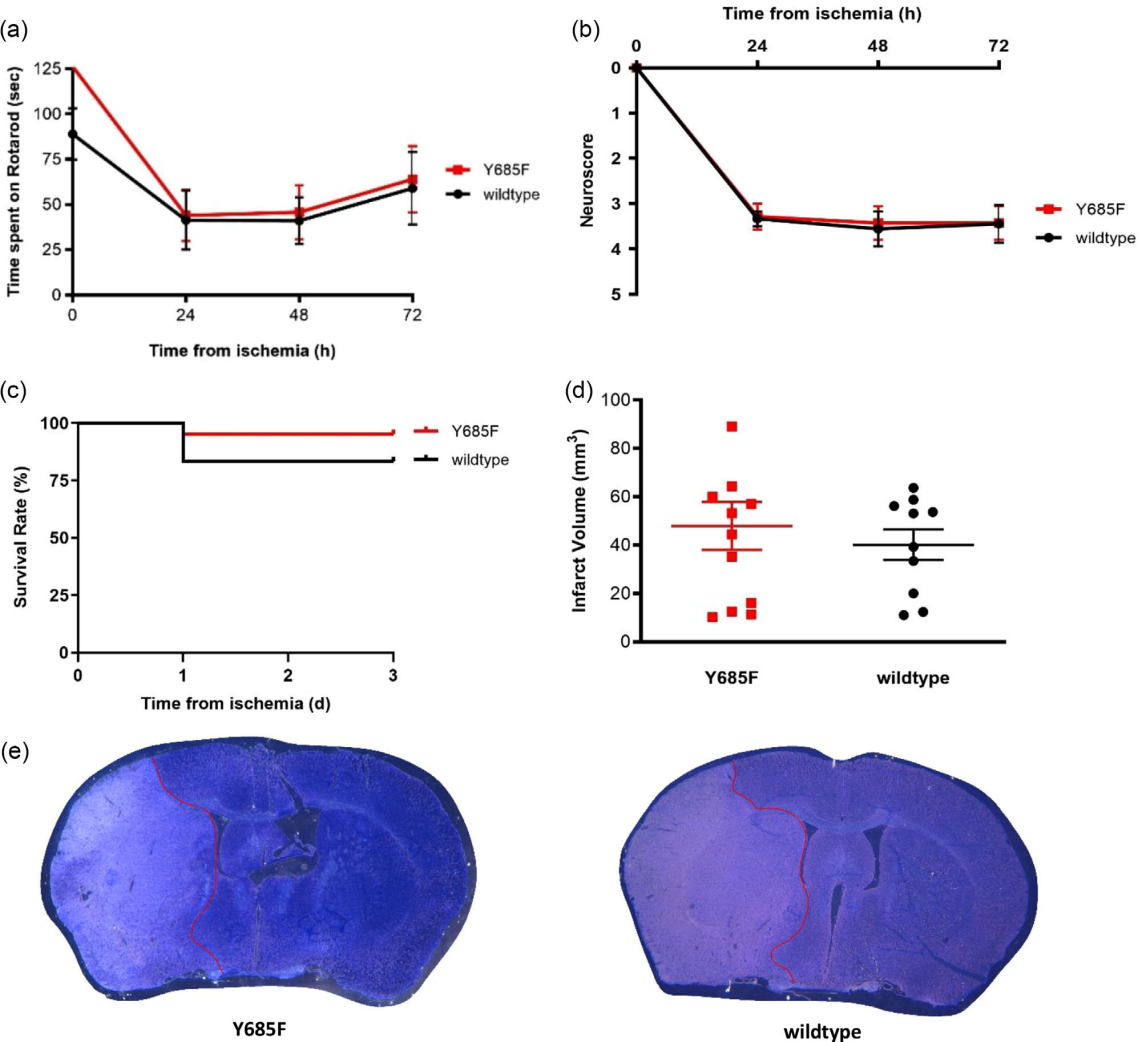
**Reduced permeability for water and macromolecules has no impact on functional and structural outcomes after ischemic stroke**. All results are displayed as mean ± SEM. (a) Rotarod test—an altered blood–brain barrier for water and macromolecules did not influence rotarod performance after an ischemic stroke. Results are presented as mean time spent on rotarod in seconds (*n* = 8, two‐way ANOVA, *p* = .5154). (b) Neuroscore—there was no difference in the neuroscore between the two groups (n = 7 and 9, two‐way ANOVA, *p* = .8889). (c) Mortality—the VE‐cadherin mutation Y685F did not impact survival curves (*n* = 21 and 18, logrank test [Mantel–Cox test] and exact Fisher test, *p* = .2279). (d) Infarct volume—the collected results showed no difference between the Y685F mice (*n* = 12) and their Wildtype littermates (*n* = 10) regarding the histological magnitude of the ischemic injury (*t*‐test, *p* = .5325). Results are a data compilation of animals that died 24, 48, and 72 h after middle cerebral artery occlusion (MCAO). (e) Toluidine staining—representative images of one of the Y7685F^d/d^ mice and one of the Wildtype littermates to visually compare the infarct volume. The infarcted area is encircled in red. There is no visual difference in the extent of the infarcted area of the Y685F^d/d^ mouse compared to the Wildtype littermate's.

**FIGURE 4:**
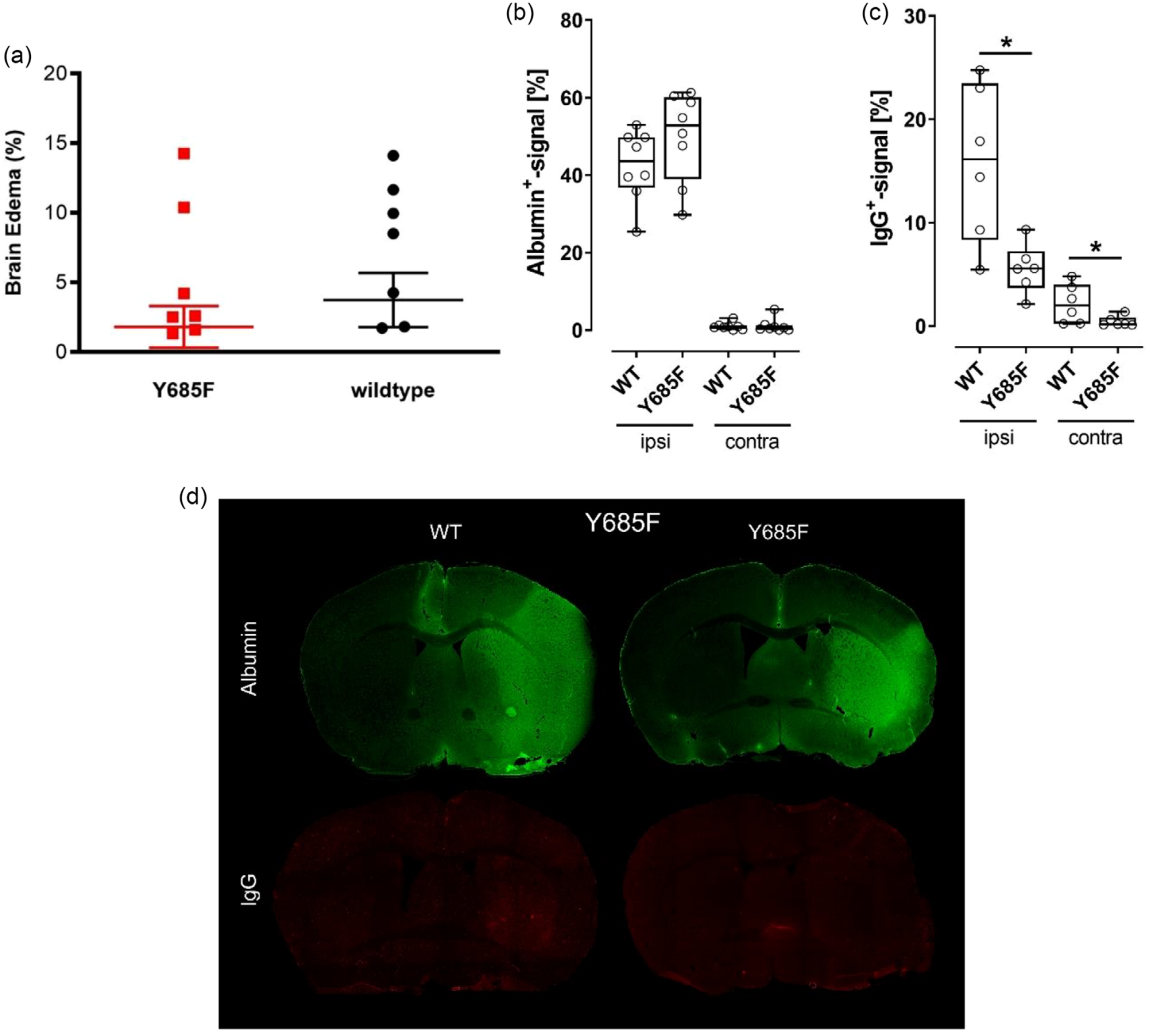
**VE‐cadherin mutation Y685F has no influence on extent of brain edema but leads to reduced IgG leakage**. (a) Extent of brain edema expressed as a percentage of the ipsilateral hemisphere did not vary between the two groups (*n* = 13 and 11, *t*‐test, *p* = .433). Results are a data compilation of animals that died 24, 48, and 72 h after middle cerebral artery occlusion (MCAO). (b) Quantification of albumin leakage—there was no difference regarding the percentage of albumin signaling between Wildtype and Y685F mice (*n* = 8; ipsilateral: *t*‐test, *p* = .181; contralateral: Mann–Whitney test, *p* = .589). The data were collected 72 h after MCAO. (c) Quantification of IgG leakage—Y685F mice showed a significantly reduced IgG leakage in the ipsilateral as well as contralateral hemisphere (*n* = 6; ipsilateral: *t*‐test, *p* = .01; contralateral: Mann–Whitney test, *p* = .041). The data were collected 72 h after MCAO. (d) Immunohistochemical visualization of albumin and IgG leakage—visually, there was no difference in the leakage of albumin or IgG between the two groups.

Although the Y731F knock‐in mouse line was used to examine the influence of altered blood–brain barrier permeability for leukocytes, the Y685F knock‐in mouse line should investigate the effect of a reduced unselective, inflammation‐associated permeability for water and macromolecules. It was shown that neither the functional outcome nor the structural outcome was influenced by this genetic modulation of the blood–brain barrier. The Y685F mice presented similar results as their wildtype littermates in respect to their motor skills and survival curves (Figure 3A, rotarod test; two‐way ANOVA, *p* = .5154, *n* = 8; Figure 3B, neuroscore; two‐way ANOVA, *p* = .8889, *n* = 7 vs. *n* = 9 and Figure 3C, survival rate; logrank test [Mantel–Cox test] and exact Fisher test, *p* = .2279, *n* = 21 vs. *n* = 18). Similarly, structural outcomes did not significantly differ between both groups. A reduced permeability for water and macromolecules did not lead to a reduced infarct volume (Figure 3D, *t*‐test, *p* = .5325, *n* = 12 vs. *n* = 10) or a diminished extent of cerebral swelling (Figure 4A, *t*‐test, *p* = .433, *n* = 13 vs. *n* = 11). The amount of albumin leakage did not differ between the two groups being compared (Figure 4B, ipsilateral: *t*‐test, *p* = .181, contralateral: Mann–Whitney test, *p* = .589, *n* = 8). IgG leakage, on the other hand, was significantly reduced in the ipsilateral as well as contralateral hemisphere of Y685F mice (Figure 4C, *p < .05, ipsilateral: *t*‐test, *p* = .01, contralateral: Mann–Whitney test, *p* = .041, *n* = 6). Figure 4D illustrates the immunohistochemical visualization of albumin and IgG leakage; no visual difference could be detected between the two groups. Altogether, these findings lead to the suggestion that humoral components are of minor relevance for the development of a secondary injury after ischemic stroke.

### 3.3 VE‐cadherin mutations do not influence the total immune cell count in ischemic brain parenchyma

**FIGURE 5:**
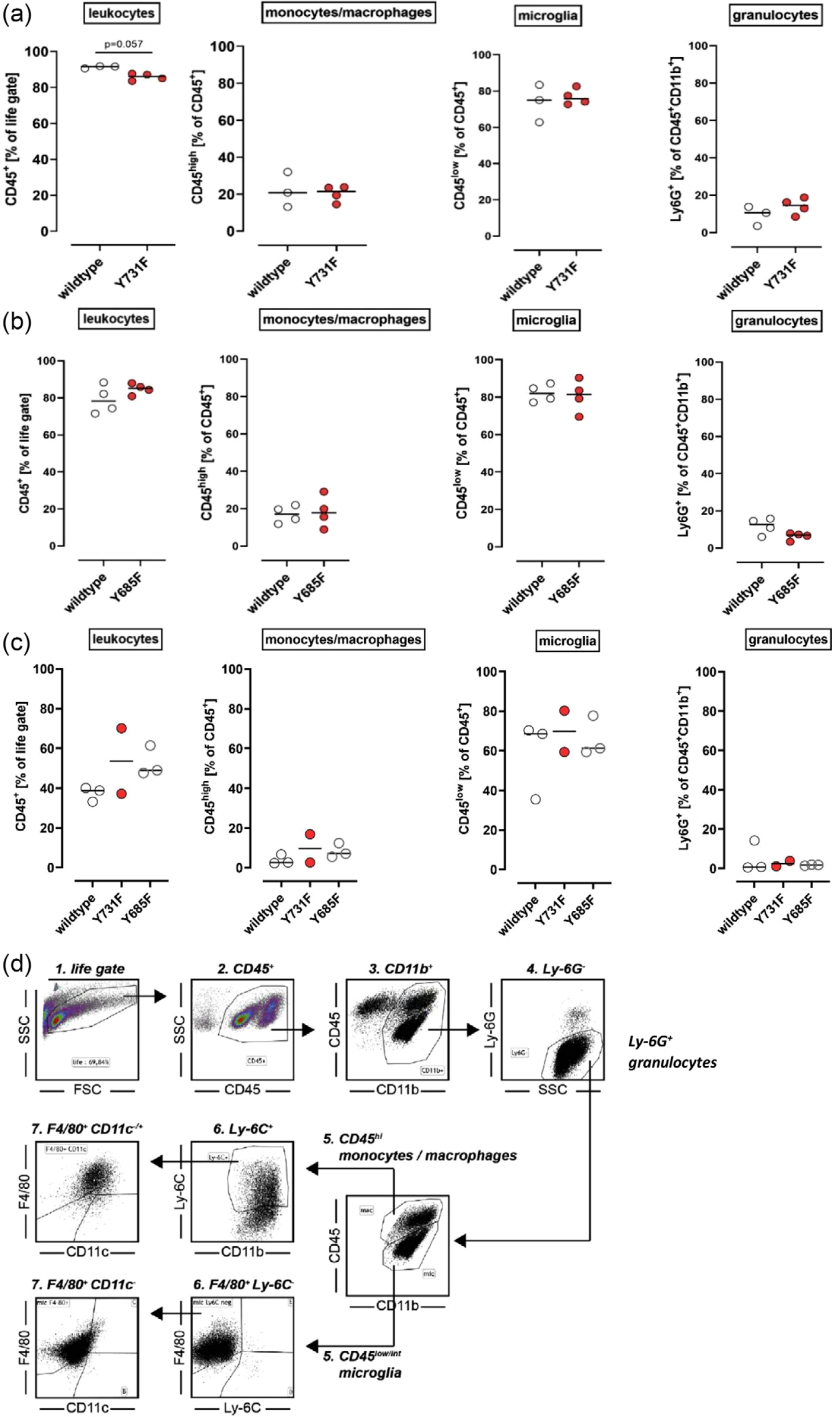
**VE‐cadherin mutations do not influence the total immune cell count in ischemic brain parenchyma**. (a) FACS analysis of Y731F mice after 72 h—after intracardial perfusion 72 h after middle cerebral artery occlusion (MCAO), murine brains were analyzed by flow cytometry. The cells were subdivided through this cell sorting mechanism into leukocytes, macrophages, microglia, and granulocytes, among others. Then frequencies of these cell types were compared between homozygotic mutant Y731F (*n* = 4) and their wildtype littermates (*n* = 3). Values are presented as median. Statistical significance was tested using the Mann–Whitney–*U* test. Not significant = not shown, *p ≤ .05. There was no difference in frequency between the groups being compared (Mann–Whitney–*U* test, leukocytes: *p* = .057, monocytes/macrophages: *p* > .999, microglia: *p* > .999, granulocytes: *p* = .4). (b) FACS analysis of Y685F mice after 72 h—after intracardial perfusion 72 h after MCAO, murine brains were analyzed by flow cytometry. The cells were subdivided through this cell sorting mechanism into leukocytes, macrophages, microglia, and granulocytes, among others. Then frequencies of these cell types were compared between homozygotic mutant Y685F (*n* = 4) and their wildtype littermates (*n* = 4). Values are presented as median. Statistical significance was tested using Mann–Whitney–*U* test. Not significant = not shown, *p ≤ .05. There was no difference in frequency between the groups being compared (Mann–Whitney–*U* test, leukocytes: *p* = .486, monocytes/macrophages: *p* = .886, microglia: *p* = .971, granulocytes: *p* = .2). (c) FACS analysis after 24 h—after intracardial perfusion 24 h after MCAO, murine brains were analyzed by flow cytometry. The cells were subdivided through this cell sorting mechanism into leukocytes, macrophages, microglia, and granulocytes, among others. Frequencies of these cell types were compared between Y865F homozygotic (*n* = 3), Y731F homozygotic mice (*n* = 2), and wildtype mice (*n* = 3). Values are presented as median. There was no difference in frequency between the groups being compared. (d) Gating strategy diagram—after defining the life gate, cells were distributed into leukocytes (CD45^+^) and non‐leukocytes (CD45^−^). All CD45^+^ cells were then divided into activated (CD11b^+^) and non‐activated (CD11b^−^) leukocytes. The next step of the gating process involved the differentiation of granulocytes (CD45^+^CD11b^+^Ly6G^+^) versus non‐granulocytes (CD45^+^CD11b^+^Ly6G^−^). To distinguish infiltrating monocytes/macrophages from resident microglia, CD45^+^CD11b^+^Ly6G^−^ cells were investigated for high and low expressions of CD45, respectively. CD45^high^Ly6G^−^ cells were defined as monocytes/macrophages, and CD45^low^Ly6G^−^ cells as microglia.

To deepen the assessment of immune cell migration after ischaemic stroke by another method, FACS analysis was applied. After defining the life gate, cells were distributed into leukocytes (CD45^+^) and non‐leukocytes (CD45^−^). All CD45^+^ cells were then divided into activated (CD11b^+^) and non‐activated (CD11b^−^) leukocytes. The next step of the gating process involved the differentiation of granulocytes (CD45^+^CD11b^+^Ly6G^+^) versus non‐granulocytes (CD45^+^CD11b^+^Ly6G^−^). To distinguish infiltrating monocytes/macrophages from resident microglia, CD45^+^CD11b^+^Ly6G^−^ cells were investigated for high and low expression of CD45, respectively. CD45^high^Ly6G^−^ cells were defined as monocytes/macrophages and CD45^low^Ly6G^−^ cells as microglia. The number of different cell subtypes was then analyzed in Y731F mice or Y685F in contrast to their littermates 72 h after MCAO. Results are given as a percentage of the corresponding gate. Y731F mice with the VE‐cadherin mutation that is supposed to result in a selective inhibition of leukocyte migration into the brain parenchyma showed a tendency to a reduced number of leukocytes, which was not statistically significant (Figure 5A, Mann–Whitney–*U* test, *p* = .057, *n* = 4 vs. *n* = 3). With regard to monocytes/macrophages, microglia, and granulocytes, no difference in numbers was found between mutants and wildtypes (Figure 5A). Same can be said about the second knock‐in mouse line Y685F. There was no difference in numbers regarding all leukocytes as well as the different subtypes monocytes/macrophages, microglia, and granulocytes (Figure 5B, *n* = 4). In addition, immune cell migration was also analyzed by FACS 24 h after MCAO. There was no difference in the number of migrated leukocytes, monocytes/macrophages, and neutrophils, as well as resident microglia among Y685F, Y731F, and wildtype mice (Figure 5C, *n* = 3 vs. *n* = 2 vs. *n* = 3).

### 3.4 VE‐cadherin mutations do not influence the spatial distribution of immune cells in ischemic brain parenchyma

**FIGURE 6:**
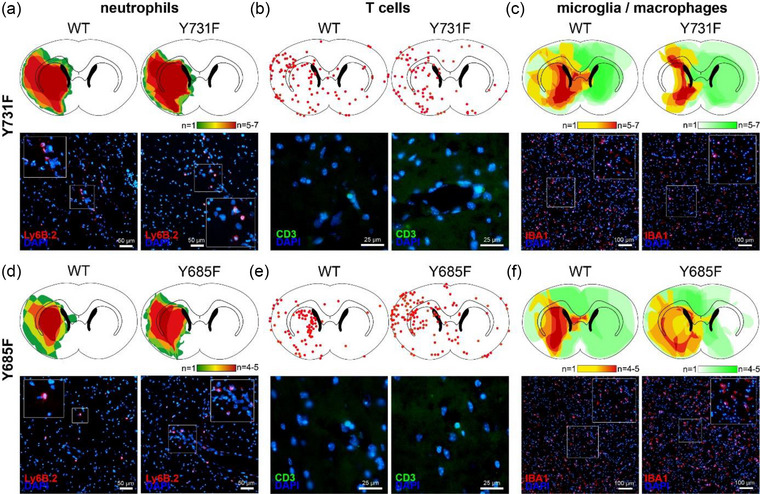
**VE‐cadherin mutations do not influence the spatial distribution of immune cells in ischemic brain parenchyma**. The right hemisphere of the murine brain is displayed on the right and vice versa. Data were collected from animals perfused 72 h after middle cerebral artery occlusion (MCAO). (a) Ly6B heatmap (Y731F)—after immunohistochemical staining with the antibody Ly6B heatmaps to show the distribution of neutrophils after ischemic stroke in mouse line Y731F were produced using a fluorescence microscope. Y731F mice (*n* = 6) were compared with their wildtype littermates (*n* = 6). There was no difference between the groups being compared. Underneath the heatmap, you can see exemplary images of the infarct area for both mouse lines photographed under the fluorescence microscope. (b) CD3 heatmap (Y731F)—after immunohistochemical staining with the antibody, CD3 heatmaps to show the distribution of T‐cells after ischemic stroke in mouse line Y731F were produced using a fluorescence microscope. Each dot represents a single cell. Y731F mice (*n* = 6) were compared with their wildtype littermates (*n* = 6). There was no difference between the groups being compared. Underneath the heatmap, you can see exemplary images of the infarct area for both mouse lines photographed under the fluorescence microscope. (c) Iba‐1 heatmap (Y731F)—after immunohistochemical staining with the antibody Iba‐1 heatmaps to show the distribution of macrophages/microglia after ischemic stroke in mouse line Y731F were produced using a fluorescence microscope. They were subclassified in dormant (green) and activated cells (red) depending on their morphological appearance. Y731F mice (*n* = 6) were compared with their Wildtype littermates (*n* = 6). It arose from the visual impression that there were less activated microglia/macrophages in the infarcted striatum of Y731F mice. Underneath the heatmap, you can see exemplary images of the infarct area for both mouse lines photographed under the fluorescence microscope. (d) Ly6B heatmap (Y685F)—after immunohistochemical staining with the antibody, Ly6B heatmaps to show the distribution of neutrophils after ischemic stroke in mouse line Y685F were produced using a fluorescence microscope. Y685F mice (*n* = 6) were compared with their Wildtype littermates (*n* = 6). It arose the visual impression that there were less neutrophils in the infarcted striatum of Y685F mice. Underneath the heatmap, you can see exemplary images of the infarct area for both mouse lines photographed under the fluorescence microscope. (e) CD3 heatmap (Y685F)—after immunohistochemical staining with the antibody CD3 heatmaps to show the distribution of T‐cells after ischemic stroke in mouse line Y685F were produced using a fluorescence microscope. Each dot represents a single cell. Y685F mice (*n* = 6) were compared with their Wildtype littermates (*n* = 6). There was no difference between the groups being compared. Underneath the heatmap you can see exemplary images of the infarct area for both mouse lines photographed under the fluorescence microscope. (f) Iba‐1 heatmap (Y685F)—after immunohistochemical staining with the antibody Iba‐1 heatmaps to show the distribution of macrophages/microglia after ischemic stroke in mouse line Y685F were produced using a fluorescence microscope. They were subclassified in dormant (green) and activated cells (red) depending on their morphological appearance. Y685F mice (*n* = 6) were compared with their wildtype littermates (*n* = 6). It arose from the visual impression that there were less activated microglia/macrophages in the infarcted striatum of Y685F mice. Underneath the heatmap you can see exemplary images of the infarct area for both mouse lines photographed under the fluorescence microscope.

To get an idea of the spatial distribution of immune cells after MCAO in our two knock‐in mouse lines, immunohistochemistry was used to make the cells visible. After immunohistochemical staining, the assessment of the cryosections under a fluorescence microscope allowed the creation of a visual impression of the spatial distribution via heatmaps. In general, it could be seen that the immune cells were predominantly located in the infarcted area of the left striatum and adjacent cortex. The Y731F mice and their wildtype littermates presented a corresponding spatial distribution of migrated neutrophils as well as T‐lymphocytes (Figure 6A,B, *n* = 6). Macrophages and microglia—which are morphologically indistinguishable as soon as the blood‐resident macrophages have migrated to the brain parenchyma—were both made visible by the antibody Iba‐1. For the visualization of the heatmaps, they were subclassified in dormant and activated cells depending on their morphological appearance. In Y731F mice, it arose the visual impression from the heatmap that there were less activated macrophages/microglia in the infarcted striatum (Figure 6C, *n* = 6). For Y685F mice with a reduced permeability for water and macromolecules, the visual impression arose that there were less activated macrophages/microglia in the infarcted striatum of Y685F mice (Figure 6F, *n* = 6).

## 4 DISCUSSION

Our results show that the mouse line Y731F with a selective inhibition of transendothelial leukocyte migration presented significantly reduced infarct volumes and a significant improvement in their motor skills. In contrast, the Y685F mutation that leads to impaired permeability for water and macromolecules had no effect on functional or structural outcomes. Both VE‐cadherin mutations seem to result in an enhanced impermeability of the blood‐brain‐barrier for IgG but not albumin.

These findings indicate that leukocytes are key players influencing both, the functional outcome and the infarct volume, whereas humoral components are of minor relevance for the development of a secondary injury after ischemic stroke.

Wessel et al. (2014) previously demonstrated in a mouse model that dephosphorylation or phosphorylation of the tyrosine residues 731 or 685 of VE‐cadherin play a crucial role in regulating the opening of endothelial cell–cell contacts. The Y685F mice in the study mentioned above showed a reduced induction of vascular permeability after intradermal stimulation with VEGF and histamine compared to wildtype and Y731F mice. In Y731F mice, however, leukocyte extravasation was significantly reduced after intrascrotal injection of IL‐1β compared to wildtype and Y685F mice (Wessel et al., 2014).

In this project, we explored for the first time the effect of these VE‐cadherin mutations in a murine stroke model. This enabled us to gain deeper insight into the consequences that these VE‐cadherin mutations have on the post‐ischemic cascade with its multiple interacting inflammatory stimuli.

Modulation of the post‐ischemic cascade for therapeutical reasons has one key advantage: The immune response to stroke develops with a delay of hours to days (Gelderblom et al., 2009; Grønberg et al., 2013), thus offering the possibility to extend the narrow therapeutic time window and thereby circumventing one of the major drawbacks of intravenous thrombolysis with rt‐PA and endovascular thrombectomy.

The components of post‐ischemic inflammation are diverse and involve leukocytes, adhesion molecules, cytokines, inflammatory enzymes such as inducible nitric oxide synthase (iNOS), and inflammatory genes such as the nuclear factor “kappa‐light‐chain‐enhancer” of activated B‐cells (NF‐κB) (Zheng & Yenari, 2004). Inhibition of leukocyte migration has been of particular scientific interest in the past. This was achieved either by cell depletion, neutralizing antibodies, chemokine inhibitors, or antagonists of adhesion molecules (Zheng & Yenari, 2004). Important representatives of the latter are the selectins, ICAM‐1, VLA‐4, and macrophage‐1 antigen (MAC‐1) (Frijns & Kappelle, 2002; Yilmaz & Granger, 2008). The results of various studies on the inhibition of immune cell migration provided inconsistent and partly contradictory results. Taking the antagonization of VLA‐4 by the antibody anti‐CD49d as an example, two studies showed a significant improvement of the structural and functional outcome (Becker et al., 2001; Neumann et al., 2015), whereas another study could not demonstrate any effect on both outcomes (Langhauser et al., 2014). Therefore, Llovera et al. (2015) wanted to investigate this in more detail in a preclinical randomized controlled multicenter trial, but they could not generate consistent results either and had to conclude that the outcome was always dependent on the type of MCAO model and the severity of the infarction. The associated clinical trials have also not shown any real success to date. Administration of natalizumab, an anti‐VLA‐4 antibody known from multiple sclerosis therapy, showed no difference between the treatment and control groups with regard to the primary endpoint, change in infarct volume from baseline determination to day 30 (Elkins et al., 2017).

An equally important component of post‐ischemic inflammation is the endothelium with its junctional proteins; after all, it forms the barrier for leukocytes and inflammatory molecules and regulates their passage into the brain parenchyma (Yu et al., 2015). It is therefore even more surprising how little research has been done to date on the importance of cell–cell contacts in stroke and how few ways of modulating them have been tested experimentally. One of the major players in this finely tuned regulatory process of barrier function and maintenance of endothelial stability is the junctional protein VE‐cadherin (Dejana & Vestweber, 2013). The alteration of the blood–brain barrier permeability for leukocytes through VE‐cadherin mutation, which was tested in this study with positive results, could offer a new therapeutical opportunity.

The use of genetically modified mice as an approach to influence the ischemic cascade is a definite strength of our study. First of all, the MCAO model in mice represents a well‐established animal model to test the effects of an ischemic stroke. This preclinical research is indispensable to develop new potential therapeutical agents. Furthermore, transgenic mice offer the opportunity to target specific genes and their modulation to study. The comparison of the transgenic mice with their wildtype littermates enables us to draw conclusion that the effect we see is to be attributed to the specific genetic modification.

The significantly reduced infarct volumes and improved motor skills of the genetically modified mouse line Y731F are most likely attributed to the attenuation of neurotoxic effects of leukocytes. Each type of leukocyte has its own selection of neurotoxic agents. Neutrophils are one of the earliest and most abundant cell types to infiltrate the ischemic brain and aggravate the cerebral injury through the production of proinflammatory cytokines like IL‐1β and TNF‐α, matrix metalloproteinases, and radical oxygen species (Gelderblom et al., 2009; Herz et al., 2015; Iadecola & Anrather, 2011; Neumann et al., 2015; Strecker et al., 2017). This leads to the destruction of the blood–brain barrier and potentially salvageable neurones in the penumbra (Heo et al., 1999; Jayaraj et al., 2019). Infiltrating macrophages release in their proinflammatory activation state cytokines like IL‐1β and IL‐6, which fuel the immune response as well as oxygen and nitrogen radicals, which have a cytotoxic effect on surrounding cells (Iadecola & Anrather, 2011; Kim & Cho, 2016; Mosser & Edwards, 2008). The secretion of IFN‐γ and perforin was detected as the two most important pathomechanisms of T‐cells in stroke. Perforin is a cytolytic protein, whereas IFN‐γ promotes the adhesion and migration of leukocytes after stroke and presumably also the progression of tissue necrosis (Liesz et al., 2011; Yilmaz et al., 2006).

A limitation of this project is the fact that, despite the significant findings in the Y731F mouse line, a reduction of intraparenchymal migrated leukocytes via FACS analysis could not be detected. There are different explanations for this:

It can be speculated that the increasing breakdown of the blood–brain barrier over time has played a crucial role. Oxidative stress and proinflammatory mediators alter the permeability of the blood–brain barrier by down‐regulating the expression of junctional proteins and thus disrupting endothelial cell–cell contacts. The consequences are the facilitated extravasation of proteins and immune cells via the paracellular route (Iadecola & Anrather, 2011). It could be seen in our project that the mutant mice Y731F and Y685F displayed a reduced leakage of IgG into the infarcted hemisphere, whereas albumin leakage showed no differences. An explanation could be that the enhancement of the blood–brain barrier impermeability through these VE‐cadherin mutations was partial and not absolute. This seems to be in accordance with the higher molecular weight of IgG (150 kDa) than albumin (66.5 kDa). In addition, it is to be known and can also be seen in our heatmaps that immune cells use alternative infiltration routes into the brain parenchyma like meninges and the choroid plexus (Benakis et al., 2018). Even though the quantification of the migrated immune cells showed no difference between knock‐in mice and wildtypes, there is another explanation that deals with the polarization of the immune cells. It is known that migrated immune cells not only assume neurotoxic but also neuroprotective functions after a stroke (Dirnagl, 2004; Iadecola & Anrather, 2011; Lo et al., 2003). The reason for this is a different polarization of the cells into a pro‐ or anti‐inflammatory activation state. This is best known in macrophages, which can perform contrary functions in an M1 or M2 activation state (Boche et al., 2013; Mosser & Edwards, 2008). M1 macrophages with the capacity for immune defense develop under the influence of proinflammatory mediators, which cause them to produce proinflammatory cytokines and oxygen and nitrogen radicals. M2 macrophages with the ability to heal wounds are created by the production of interleukin‐4, whereupon they produce components of the extracellular matrix to repair the injury (Boche et al., 2013; Mosser & Edwards, 2008). It is conceivable that the Y731F point mutation has led to a reduced influx of proinflammatory activated immune cells, shifting the balance toward an anti‐inflammatory state. This could provide an explanation for the reduced infarct volumes and improved motor skills despite lacking differences in the FACS analysis.

Furthermore, rodent stroke models and even more stroke models using genetically modified mice have their limitations, making a translation to human stroke pathology possible. It is unsure what effect the VE‐cadherin mutations have in other organs. But this also applies to other models of inhibition of leukocyte migration such as chemical or antibody‐mediated leukocyte depletion.

A great strength of our work is that we see a positive effect on both functional and structural levels. It is particularly important to emphasize that this is a targeted improvement in neurological outcome and not only an effect that can be explained by a better general condition. Y731F mice show significantly better results in the rotarod test while presenting no difference in survival analysis or neuroscore when compared to their wildtype littermates.

So far, we are the first to use these mouse lines in a stroke model. Further studies are needed to evaluate the effects in other organ systems. In principle, however, modification of the post‐ischemic cascade via VE‐cadherin appears to be a potentially effective target for the development of future stroke therapies.

In conclusion, we could show that an alternative model of reduced leukocyte migration by VE‐cadherin mutation after murine ischemic stroke leads to smaller infarct volumes and improved motor skills. VE‐cadherin thus appears to be a potential target for the development of future pharmacological therapies.

## AUTHOR CONTRIBUTIONS

Antje Schmidt‐Pogoda and Jens Minnerup planned and designed the project. Carolin Beuker and Frederike Anne Straeten contributed to project related discussions. Antje Schmidt‐Pogoda and Mailin Hannah Marie Koecke carried out motor testing of mice. Antje Schmidt‐Pogoda and JKS performed surgical procedures. MHMK carried out immunohistochemical stainings. Antje Schmidt‐Pogoda, Jan‐Kolja Strecker, and Mailin Hannah Marie Koecke analyzed data for structural and functional outcomes as well as immunohistochemistry. Jan‐Kolja Strecker and Anna‐Lena Börsch carried out the FACS analysis. Antje Schmidt‐Pogoda, Jan‐Kolja Strecker, and Mailin Hannah Marie Koecke created the figures and wrote the manuscript. All authors read and approved the final manuscript.

## CONFLICT OF INTEREST STATEMENT

The authors declare no conflicts of interest.

### PEER REVIEW

The peer review history for this article is available at https://publons.com/publon/10.1002/brb3.3449.

## Data Availability

All original data will be made available upon reasonable request.
